# Marek’s disease virus and skin interactions

**DOI:** 10.1186/1297-9716-45-36

**Published:** 2014-04-03

**Authors:** Mathilde Couteaudier, Caroline Denesvre

**Affiliations:** 1 INRA, UMR1282, Infectious Diseases and Public Health, ISP, BIOVA team, F-37380 Nouzilly, France

## Abstract

Marek’s disease virus (MDV) is a highly contagious herpesvirus which induces T-cell lymphoma in the chicken. This virus is still spreading in flocks despite forty years of vaccination, with important economical losses worldwide. The feather follicles, which anchor feathers into the skin and allow their morphogenesis, are considered as the unique source of MDV excretion, causing environmental contamination and disease transmission. Epithelial cells from the feather follicles are the only known cells in which high levels of infectious mature virions have been observed by transmission electron microscopy and from which cell-free infectious virions have been purified. Finally, feathers harvested on animals and dust are today considered excellent materials to monitor vaccination, spread of pathogenic viruses, and environmental contamination. This article reviews the current knowledge on MDV-skin interactions and discusses new approaches that could solve important issues in the future.

## Table of contents

1. Introduction

2. Marek’s disease virus

3. Pathophysiology of Marek’s disease

4. Chicken skin and feather follicles

4.1. Chicken skin structure

4.1. The feather follicle: the organ that generates feathers

5. Feather follicles support the excretion and horizontal transmission of MDV

6. Methods for MDV detection in the skin or feathers (diagnostic methods)

7. MDV replication in feather follicles

8. MDV tropism for feather follicles - hypotheses

9. Impact of host genetics on MDV replication in the skin

10. Cutaneous lesions after MDV infection

11. Atypical morphogenesis of MDV in the skin

12. Viral molecules associated with MDV replication in feather follicles or with the infectiousness of particles excreted from the skin

13. Excretion of vaccinating strains and pathogenic strains of MDV

14. Immune response in the skin of MDV-infected chickens

15. Conclusions

16. Abbreviations

17. Lexicon

18. Competing interests

19. Authors’ contributions

20. Acknowledgements

21. References

## 1. Introduction

Marek’s disease virus (MDV), or Gallid herpesvirus 2 (GaHV-2) is the etiological agent responsible for Marek’s disease (MD) in the chicken, a multifaceted disease most widely recognized by the induction of a rapid and extensive malignant T-cell lymphoma. MD has been shown to occur worldwide according to data from the world Organization for Animal Health (OIE), although data are difficult to obtain because this disease is not a notifiable disease. MD results in substantial economic losses, estimated at more than 1 billion per year [1]. Although MD was described in 1907 by Joseph Marek, the virus (MDV) was only isolated in 1967 in the United Kingdom [2] and the United States [3] independently. MDV belongs to the family of *Herpesviridae*, the subfamily of *Alphaherpesvirinae*, and the genus *Mardivirus* (for Marek’s disease-like viruses). MDV was initially classified within the *Gammaherpesvirinae* due to its biological properties, but was reclassified in 2002 (after the complete sequencing of its genome) in the new *Mardivirus* genus, for which it became the type-species [4]. To date this genus comprises 4 other species: the Gallid herpesvirus 3 (GaHV-3), the Meleagrid herpesvirus 1 (MeHV-1) - commonly known as herpesvirus of turkey (HVT), the Anatid herpesvirus 1 and the Columbid herpesvirus 1. GaHV-3 and HVT infect domestic fowls like MDV, but are not pathogenic.

MDV is the first oncogenic virus for which an effective vaccine has been developed, in the late sixties [5-7]. In the early seventies, when large scale vaccination started in poultry houses, MDV was responsible for a large mortality and morbidity. Since this time, vaccination has allowed the thriving industrial production of eggs and poultry meat. All the currently used vaccines are live vaccines derived from the three viral strains: the HVT FC126 strain [7], the GaHV-3 SB-1 strain [8], and the GaHV-2 CVI988/Rispens strain [9]. HVT and SB-1 vaccines are considered heterologous vaccines because they are derived from a different viral species than the virus it is intended to protect against, while the Rispens vaccine is considered homologous because it is from the same viral species as the targeted virus.

## 2. Marek’s disease virus

Herpesvirus infectious particles comprise more than 30 different proteins, assembled according to a complex architecture including the following: (i) a central capsid containing the viral genome, (ii) a protein layer termed tegument, comprising more than 15 proteins, and (iii) a lipid bilayer in which about 10 envelope glycoproteins are anchored. The MDV genome is a linear double-stranded DNA of approximately 175 kb, which contains a unique long (UL) sequence and a unique short (US) sequence, both flanked with terminal repeat (TR) and internal repeat (IR) sequences [4]. Owing to its structure, this genome belongs to group E, like the human herpesvirus 1 (HHV-1). The MDV genome contains about 100 open reading frames and encodes more than 70 genes, most of which have orthologous equivalents in other alphaherpesviruses (e.g., tegument genes like UL36 [VP1/2], the largest ORF in the genome, UL47 [VP13/14] and UL49 [VP22] or capsid genes like UL19 [VP5]) [4]. However, some genes are specific to MDV, such as the gene encoding Meq oncoprotein or pp38 phosphoprotein [4].

To date, MDV replicates efficiently only in primary chicken or duck cells in culture [2,3], yielding titers between 10^5^ and 10^7^ pfu/mL depending on the strain. MDV infections are performed by co-culturing infected cells with naïve cells because the virus cannot be purified as cell-free virus from cell lysates or culture supernatants. These different characteristics constitute constraints for vaccine production.

## 3. Pathophysiology of Marek’s disease

**Figure 1:**
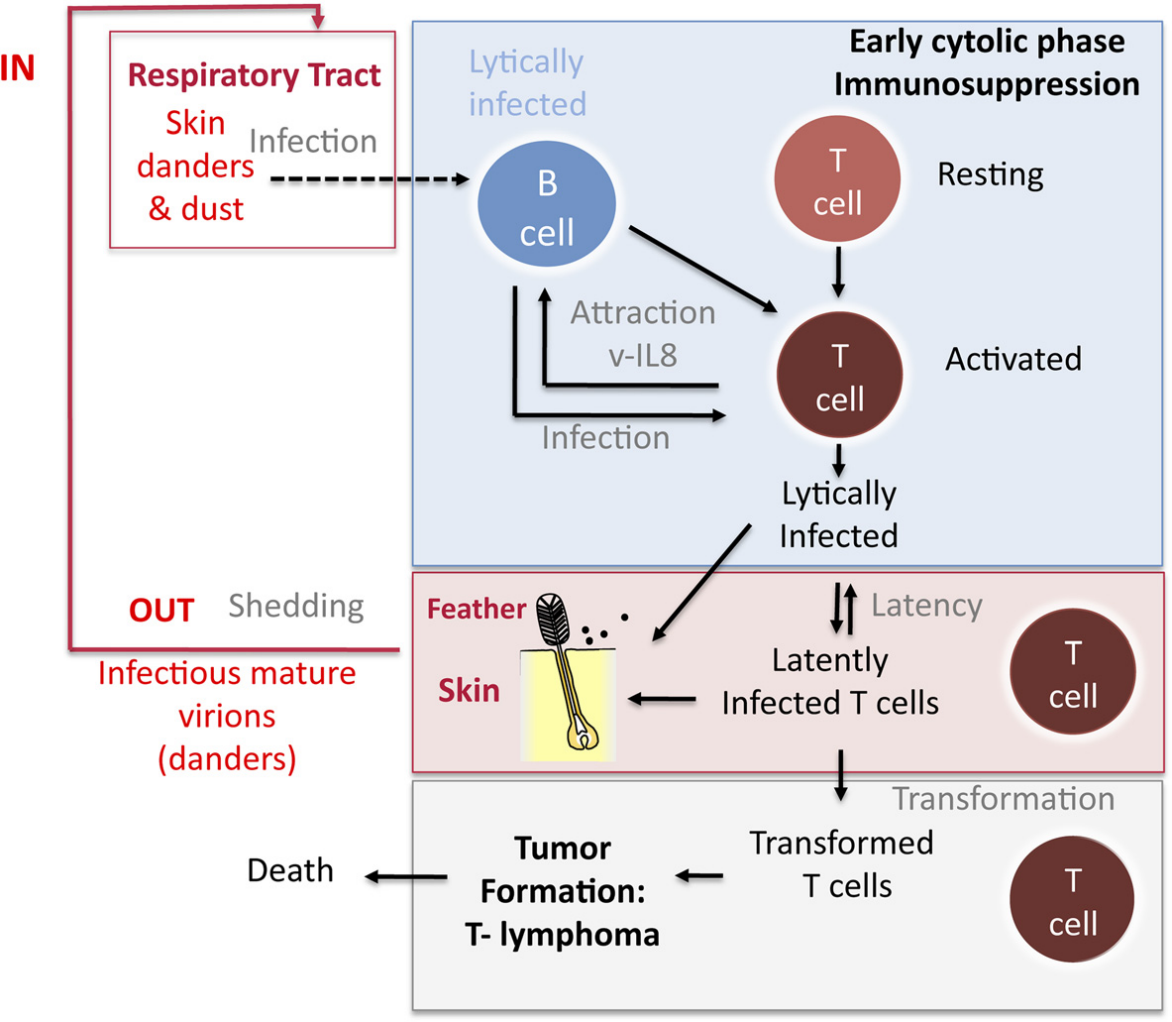
**Pathophysiology of Marek’s disease (adapted from Calnek model [**[10]**,**[11]**]).** Marek’s disease virus (MDV) enters into the chicken through the respiratory tract. MDV has a tropism for B- and T- lymphocytes as well as for the feather follicle epithelium, from which MDV is shedded into the environment. Feathers, skin danders and dust are the major source of MDV infectious materials and the basis of horizontal bird-to-bird transmission in field conditions.

The current model of MD pathophysiology was initially proposed by Bruce Calnek [10,11]. This model is described in Figure 1. MDV enters via the chicken respiratory tract after inhalation of contaminated dust. Then MDV infects B lymphocytes and macrophages in the lungs [12] and is then transported towards the main lymphoid organs (bursa of Fabricius (see lexicon), thymus, and spleen). After replicating in B lymphocytes, MDV infects activated T lymphocytes, mainly CD4+ cells. It is believed that only a few T lymphocytes undergo transformation and are at the origin of the T lymphoma, which may be either monoclonal or oligoclonal [13]. This lymphoma is mostly localized in visceral organs (kidneys, spleen, liver, gonads, and proventriculus), peripheral nerves, skin, and muscles. In most transformed T lymphocytes, the virus is in latent phase and does not produce viral particles. Only a small proportion of tumor cells (< 0.01%) expresses lytic viral antigens and contains viral particles detectable in transmission electron microscopy (TEM) [14]. Of note, MDV only enters latency in lymphocytes but not in neurons, like most alphaherpesviruses. Early during infection, the virus is transported towards the skin, most specifically to feather follicles. From infected feather follicles, MDV is shed into the environment via scales and feather debris, which become the major source of contamination of other birds in the natural environment. Bird-to-bird transmission is exclusively horizontal. There is no vertical transmission from the chicken to the egg, even though the embryo can be experimentally infected [15]. In typical housing conditions, it is believed that animals become contaminated at a young age. MDV interactions with chicken skin is considered the major cause of MDV persistence in poultry houses and its evolution towards increasingly more virulent genotypes has been observed for the past decades [16,17]. To this end, in this review we present the current state of knowledge of MDV interactions with chicken skin. For other aspects of MDV biology, we refer the reader to other reviews [18-20].

## 4. Chicken skin and feather follicles

### 4. 1. Chicken skin structure

**Figure 2:**
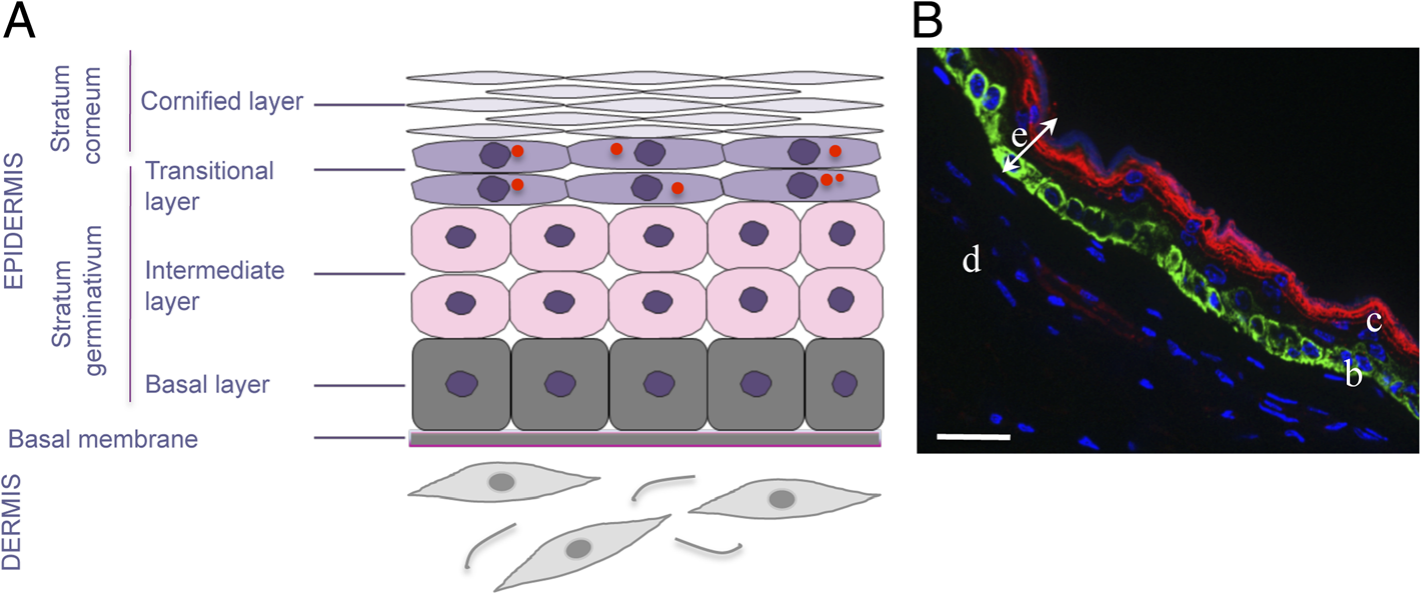
**Structure of chicken apteric skin. (A)** Schematic chicken skin section without feather follicle. The epidermis is constituted of four layers with keratinocytes at various differentiation stages. The red circles represent lipid droplets. **(B)** Expression and localization of two markers of the epidermis, involucrin (red) and keratin 14 (green), detected by immunofluorescence. The skin was harvested from a 53-day old white leghorn chicken, fixed in 4% paraformaldehyde, frozen and embedded in cryomatrix compound. Seven μm-thick cryosections were stained with two antibodies: anti-keratin 14 (green) and anti-involucrin (red). Secondary antibodies used were conjugated to Alexafluor 488 or 594. The nuclei were stained with Hoechst 33342 dye (blue). Images were captured on an Axiovert 200 M inverted epi-fluorescence microscope in the presence of the ApoTome system (Zeiss, Göttingen, Germany). d: dermis; e: epidermis; c: cornified layer; b: basal layer. Bar, 20 μm.

**Figure 3:**
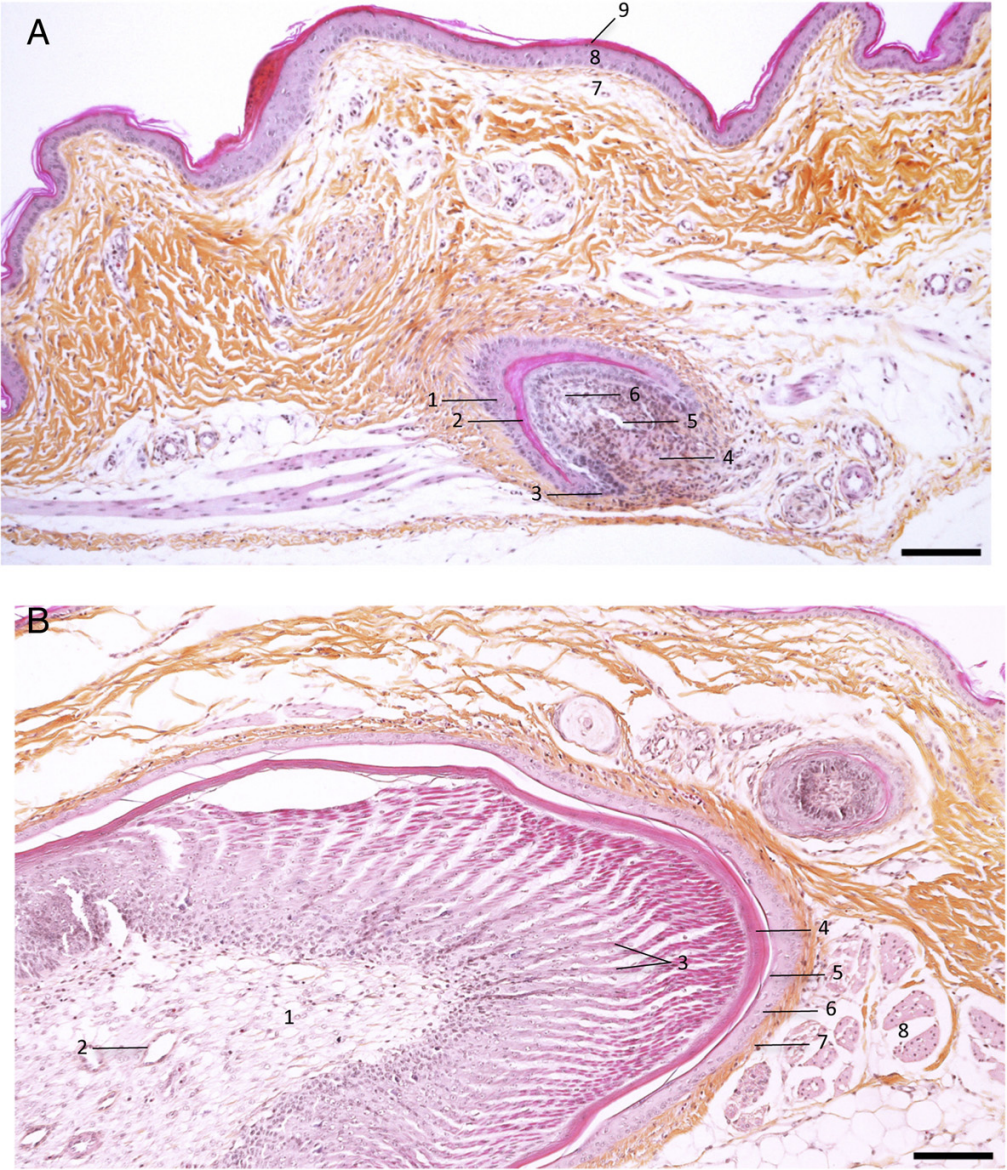
**Chicken skin with feathers.** The skin from a 53-day old white Leghorn chicken, was harvested, fixed in 4% paraformaldehyde, and embedded in paraffin. Sections were stained with hematoxylin, eosin, saffron. **(A)** Skin region with a feather follicle. Feather follicle (1 to 6); skin (7 to 9): 1, feather follicle; 2, cornified cells; 3, epidermal collar; 4, dermal papilla; 5, axial blood vessel; 6, Feather pulp; 7, Dermis of the skin; 8, stratum germinativum of the skin; 9, stratum corneum of the skin. **(B)** A growing feather: 1, feather pulp; 2, axial blood vessel; 3, barbs; 4, feather sheath; 5, stratum corneum (follicle); 6, stratum germinativum (follicle); 7, Dermis; 8, muscle feather. Bar, 100 μm. (Pictures kindly provided by T. Larcher).

In vertebrates, the skin is the first layer of protection against the external environment. The skin plays an important role in thermal, hygrometric, and chemical regulation. Bird’s skin differs from that of mammals by its thinness, by the presence of feathers instead of hair, and by the absence of sebaceous glands, although the overall histological structure is similar [21,22]. Bird’s skin is composed of an epidermis separated from a dermis by a basal membrane (Figures 2 and 3). Table 1 presents the cell markers mentioned in this review that are used to characterize the various skin layers; these markers are generally defined by their homology to that of mammals, based on their DNA sequence. The basal membrane is a thin and continuous layer which serves as a molecular filter and anchoring point for the epidermis basal cells via hemidesmosomes. This extracellular matrix is mainly constituted of type IV collagen and proteoglycans. Bird dermis is relatively thin compared to that of mammals. It is mainly constituted of connective tissue arranged in a superficial layer (or *stratum superficiale*) and a deep layer (or *stratum profundum*). The dermis can be identified by the expression of cell markers such as fibronectins. The epidermis is a multistratified, keratized squamous epithelium, whose thickness varies depending on the region of the body. The epidermis deep layer (*stratum germinativum*) is composed of live cells arranged in three layers: the basal, intermediate, and transitional layers (Figure 2). The basal layer, which is next to the basal membrane, is constituted of small undifferentiated cubic cells, which have a high dividing rate and which migrate towards more superficial layers. The basal layer can be identified with cell markers such as basonuclin 2, keratins 5 and 14 [23,24] (Figure 2B). The intermediate layer is constituted of cubic cells that have migrated from the basal layer. The bird’s intermediate layer is similar to mammal’s spinous layer. The intermediate layer can be detected via the expression of transglutaminase 5 or desmoglein 2 [25]. The transitional layer is constituted of two or three layers of flat elongated cells containing a large number of intracellular lipid vacuoles or droplets, which is typical of bird’s skin. This layer expresses keratin 10 and 75 (alpha-keratin KIIB) [24,26]. The external layer of the epidermis or cornified layer (also called *stratum corneum*) is composed of corneocytes, which are flat dead anucleated keratinized cells organized in sheets. This layer can be identified by the presence of involucrin, loricrin, or filaggrin [27] (Figure 2B). The differentiation of basal cells in corneocytes is a normal physiological process in the epidermis. The main cellular modifications are the loss of organelles, the formation of lipid vacuoles and keratin fibers in the cytoplasm and a thick envelope under the plasma membrane [22]. Corneocytes, which detach regularly from the epidermis, are constantly renewed by the cells from the lower layers. This process called exfoliation or desquamation results from the loss of desmosomes between corneocytes.

**Table 1:** Cellular markers of chicken skin

**Cell layers of the skin**	**Cellular markers**	**Functions**
**Dermis**	Fibronectin	Glycoprotein which contributes to extracellular matrix organization and favors cell adherence
**Basal membrane**	Laminin	Protein complex, an essential component from the basal membrane, which constitutes a molecular filter
**Basal layer of epidermis**	Basonuclin 2	Nuclear protein with zinc fingers, transcription factor which maintains the proliferative ability and prevents the terminal differentiation
	Keratin 5 (KRT5)	α-keratin type II, intermediate filament of proliferating keratinocytes, associated to KRT14
	Keratin 14 (KRT14)	α-keratin type I, intermediate filament of proliferating keratinocytes, associated to KRT5
**Intermediate layer of epidermis**	Transglutaminase 5	Calcium dependent-enzyme involved in the first differentiation steps of the epidermis
	Desmoglein 2	Cadherin playing a role in the formation of desmosomes, that connect together epithelial cells
**Transitional layer of epidermis**	Keratin 75 (KRT 75)	α-keratin type IIB, marker from the terminal differentiation of the keratinocytes
	Keratin 10 (KRT10)	α-keratin type I, marker from the terminal differentiation of the keratinocytes, associated to keratin 1
**Cornified layer of epidermis**	Involucrin	Precursor protein from the corneocyte envelope
	Filaggrin	Basic protein associated to keratin filaments of the cornified layer
	Loricrin	Precursor protein from the corneocyte envelope

As in mammals, chicken epidermis contains dendritic cells (Langerhans cells), whose number is estimated at 8000 per mm^2^ of epidermis in an 8-week chick [28,29]. These two studies were conducted in the apteric areas (see lexicon) of the skin that have no feathers. Following antigenic stimulation, these cells seem to migrate to dermal lymphoid nodules, and not to lymph nodes that are absent in birds [29]. Besides feathers, bird’s epidermis contains melanocytes, including in non-colored chickens. The “silky-chicken” strains, which have a dark skin and white or black feathers (“white silky” or “black silky”), are the only strains that also have a large number of melanocytes in the dermis and in the connective tissue of deep organs [30].

### 4. 2. The feather follicle: the organ that generates feathers

Birds are the only animals for which feathers are absolutely necessary for flying but also act as a thermal barrier. Feathers are the most complex and most diversified integumentary products found in vertebrates. Feathers are exclusively constituted of ß-keratin [31] and arise from the feather follicle. The feather follicle forms by invagination of the epidermis around the feather filament cylinder into the dermis, at day 14 of embryogenesis, which lasts 21 days in chickens [32]. There are as many feather follicles as there are feathers on the skin, i.e., between 20 000 and 80 000 depending on the bird species [32]. At the base of the feather follicle are located the dermal papilla, the epidermal collar and the collar bulge (Figure 3). Follicle stem cells, which are located in the collar bulge, give rise to a population of transient amplifying (TA) cells, which allow the renewal of the feather and the follicle after molting or after accidentally plucking the feather [33,34]. Repeated molting ensures the regular renewal of bird feathers throughout its lifespan.

Feather follicles contain melanocytes responsible for the color of the feathers, as well as melanocyte stem cells, which were recently identified by the Chuong laboratory [35]. In a regenerating follicle, melanocyte stem cells (pigmented or not) are located in the epithelium, above the dermal papilla, in the lower part of the bulge. In a resting feather follicle, melanocyte progenitors move into the dermal papilla, where they remain quiescent [35].

At the feather level, pulp cells originate from the dermal papilla cells, while all other cells derive from the epidermal collar and the collar bulge [32]. The base of the feather is vascularized by an arteriole which goes through the dermal papule and the pulp of the feather (Figure 3).

## 5. Feather follicles support the excretion and horizontal transmission of MDV

It has been known since 1963 that, in natural conditions, disease transmission is airborne [36,37], suggesting that the virus is excreted and relatively resistant in the external environment. Moreover, the observation of cutaneous lesions in birds with MD and the detection of MDV antigens via immunofluorescence in feather follicles led early on to the suspicion that feather follicles were involved in the excretion of the virus [38]. In 1970, it was shown that dust, scales, and feather debris collected in infected poultry houses could lead to MD after intra-abdominal administration to chicks or after introduction in the confined environment of healthy chickens [39,40]. The presence of infectious virions in the skin and feather follicles of infected chickens was confirmed a few months later by the teams of Calnek and of Nazerian [41,42]. To this end, skin or feather tip homogenates of infected chickens were observed using negative TEM. When administered to healthy chickens this material was capable of reproducing MD. These findings demonstrated that the feather follicle can produce complete mature infectious virions, harboring a tegument and an envelope. Still today feather follicles constitute the only biological material that allows the extraction of enveloped infectious virions and transmission of the infection in the absence of associated cells. The infectiousness of MDV in the environment can last up to 7 months at room temperature [43] and 16 weeks in litters [44], a duration that is unusual for a herpesvirus. These findings suggest that infectious viral particles are probably not in direct contact with the environment but physically protected from degradation, possibly by cellular material (see the section regarding viral morphogenesis below).

## 6. Methods for MDV detection in the skin or feathers (diagnostic methods)

In this paragraph we will only cite the methods that were applied to MDV detection in the skin and/or feathers. Until the 1980s, these methods were aimed at detecting viral antigens by immunofluorescence on tissue sections [38], or by gel immunodiffusion or ELISA from feather tip cell extracts [45,46]. In the 1960s and 70s, these antigens were detected using the serum of infected chickens. Today, polyclonal serums and monoclonal antibodies against single viral proteins are also available. TEM has been used to visualize viral particles in situ in the skin or in tissue extracts (see section on viral morphogenesis below). Since the 1990s, new methods based on molecular biology techniques have appeared, enabling mardivirus genome detection (PCR) [47,48] and quantification (qPCR) [49-52]. It is also possible to detect viral DNA in feathers by pulse field gel electrophoresis (PFGE) [53] or via in situ hybridization [54]. An inexpensive and rapid method of amplification of the viral genome called LAMP (loop-mediated isothermal amplification) has been recently developed to allow rapid diagnostic in field conditions [55]. The substantial improvement of sequencing techniques has also allowed the direct sequencing of viral DNA extracted from feather tips to detect coinfections for instance [56]. Moreover, PCR methods allow the detection and quantification of viral DNA in dust collected and concentrated on filters [51,57,58]. Finally, MDV can also be re-isolated from feather pulp via co-culture in vitro [14]. To this end, the pulp is extracted from the base of the feather, digested using collagenase, and the resulting cell suspension is incubated with a monolayer of permissive cells.

For the past years, feathers and dust have been considered the material of choice to follow the evolution and distribution of pathogenic and vaccine strains of Mardiviruses in poultry houses [59]. Four to five pulp-rich feathers, preferably collected on the axillary tract, are sufficient to detect viral DNA using qPCR (S. Baigent personal communication).

## 7. MDV replication in feather follicles

**Figure 4:**
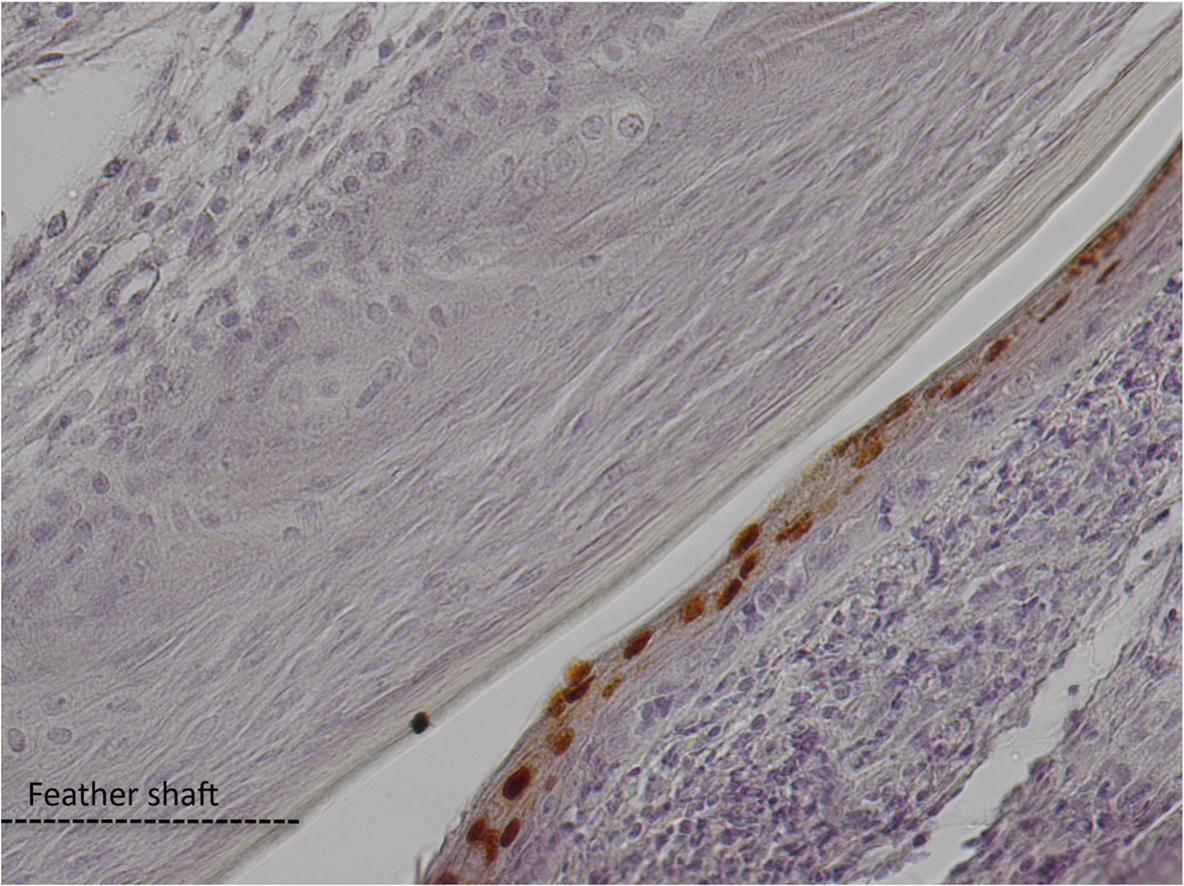
**Detection of VP5 major capsid antigen in MDV infected feather follicle by immunohistochemistry.** The VP5 protein is located in the nuclei of the upper layers from the feather follicle epithelium (brown nuclei), at the junction with the feather shaft. (Picture kindly provided by J-F. Vautherot).

Regarding viral antigen expression, the epithelium of feather follicles is the tissue the most commonly found positive in infected chickens, compared to other tissues [38,45]. It is also the infected tissue that expresses the highest level of viral antigens for the longest period of time. These antigens are located in the upper layers of the *stratum germinativum* of feather follicles (Figure 4). Viral antigens are detectable in feather follicles from feathers tips 11 to 14 days post-infection (pi) using standard biochemical methods [60,61]. With more sensitive methods such as qPCR, viral DNA can be detected as early as 6–7 days pi in feather tips and in dust collected in isolation units [42,62,63]. A recombinant virus encoding the tegument gene UL47 fused with mRFP (monomeric Red Fluorescent Protein) allows the detection of lytic viral infection in feather follicles using fluorescence as early as day 8 pi [63]. The difference between the detection of the viral genome and its expression is due either to the difference in method sensitivity or to the delay between viral replication and the accumulation of late viral proteins to a sufficient level. The kinetics of replication of mardiviruses in feathers has been found to vary depending on the virus strain [63]. These variations do not seem to be directly linked to the strain’s virulence, as it was formerly believed [42]. In fact, non-virulent strains can be detected in feathers and dust as early as highly virulent strains, and can even be excreted at higher levels [62,64]. It is noteworthy that excretion of MDV strains increases considerably from 7 to 28 days pi, reaching a plateau thereafter, according to quantitation experiments of viral genomes conducted on dust in isolation units [62]. Moreover, there is a strong correlation between the quantity of the MDV genome measured in feathers and dust [57].

Coinfection of birds with two pathogenic strains (regardless of their similarity of genotype or pathogenicity) leads to the replication of both viruses within the same feather follicle. This was demonstrated in several studies on feather follicle sections using fluorescence or immunohistochemistry utilizing viruses that have different antigenic markers or expressing different fluorescent reporter genes (e.g., GFP and mRFP) [56,65]. Jarosinski also showed that two fluorescent viruses with the same genotype can infect the same feather follicle cell [65]. This suggests that genetic recombinations between two different genomes could occur in the feather follicle to yield new strains. However, analysis of the frequency and distribution of two viral genomes after coinfection at different times pi by pyrosequencing has shown that some strains may preferentially replicate in feather follicles when compared to other strains [56].

## 8. MDV tropism for feather follicles - hypotheses

The mechanisms by which MDV infects skin and feather follicles are poorly understood. Because B and T lymphocytes are the major targets of MDV and are infected early on [10,12], it is probable that these cells are the vehicle to feather follicle infection. However, this has not been formally demonstrated; therefore the involvement of other blood cells (e.g., macrophages and/or dendritic cells) cannot be excluded. In addition, for most pathogenic strains, replication starts at 1 week pi in the feather follicle, well before tumor development. Therefore, it is probable that it is not transformed cells that migrated into the skin, as at this time, there are no or very few transformed cells.

Regarding how the virus reaches the transitional layer of the feather follicle epithelium, many questions remain unanswered: Why is the virus mainly present in the epidermis of feather follicles and not in the epidermis of the whole skin? Is the epidermis infected directly or indirectly, via the dermis? Does the virus directly infect the epidermis upper layers, or does it enter the basal layer first and then replicate only when those differentiate? How does the virus cross the basal membrane?

Various speculative scenarios can be proposed: (i) “cargo” infected cells (lymphocytes or other) infiltrate the skin epithelium to transmit the virus to the upper epithelial cells of the epidermis, and the virus propagates to other neighboring cells and so on; or (ii) lymphocytes infiltrate the dermis or the dermal papilla, infects neighboring cells such as fibroblasts or melanocyte precursors, which in turn transmit the virus to the basal epithelial cells of the epidermis, in which case it requires MDV to cross the basal membrane; or (iii) lymphocytes directly infect the follicle stem cells located in the bulge of the feather follicle, and the infection spreads widely to TA cells (see section on feather follicle above) that are involved in the repair of the follicle wall and the feather during feather regeneration, a process that occurs frequently at a young age. The development of new techniques and methods such as transgenic chickens harboring fluorescent transgenes in specific cell lineages (lymphocytes or dendritic cells for instance), methods enabling the in vitro culture of chicken skin that mimic a multilayer epithelium, and biphotonic imaging on thick tissue should help answer these questions in the near future.

## 9. Impact of host genetics on MDV replication in the skin

All lines of *Gallus gallus*, including exotic ones [66,67], seem susceptible to MDV infection. Interestingly, in poultry houses, MD similarly affects chicken breeds for meat production and those for egg production, even though these two types of productions may not be equally affected in some countries due to breeding practices. Although some chicken genetic markers have been shown to be involved in the susceptibility or resistance of chickens to tumors [68,69], no marker so far has been shown to regulate viral production in the epithelium of feather follicles. Further research in this area may help reduce or block the excretion and spreading of pathogenic MDV strains.

Two chicken lines with mutations that affect their normal skin physiology, have particular patterns of skin interaction with MDV that merit attention. The first line is the “scaleless” line, which carries a recessive autosomal mutation *sc* (for “scale”) and produces “naked” chickens lacking scales on their legs and harboring only a few sparse feather follicles. Administration of skin cell extracts from scaleless chickens 29 days after infection with a hypervirulent strain (686) to naive chickens indicates that epithelial cells not associated with feather follicles are capable of transmitting infection and producing infectious viral particles [70]. In that study, however, no result was presented regarding the ability of these birds to transmit MD horizontally to susceptible chickens or to chickens of the same genotype to determine whether these animals excrete infectious virions in the environment.

The second line is the Smyth (SL) line, which has colored feathers similarly to the Brown Leghorn; this line spontaneously develops an autoimmune disease leading to a depigmentation of regenerating feathers, which become white due to the death of melanocytes. This line is considered as an animal model for human vitiligo [71]. The depigmentation of SL birds occurs between 6 and 14 weeks of age in 70% to 95% of the birds. When birds were moved from one university to another, the phenotype was only observed in 10% of the population, suggesting the role of the environment in addition to genetic factors. To determine the reasons for this difference, the environment and breeding conditions in the two animal facilities were compared. Among three important differences, vaccination of the birds against MD using the heterologous HVT appeared to be the most important factor. Indeed, it has been shown that 20 week-old birds vaccinated with HVT had an incidence of vitiligo 4 times higher than non-vaccinated birds [72]. This puzzling result raises various hypotheses regarding the impact of HVT vaccination on the development of vitiligo, knowing that the HVT vaccine also penetrates and replicates in feather follicles [51,59]. In the SL line, depigmentation is associated with melanocyte death and with the presence of anti-melanocyte auto-antibodies; therefore, one hypothesis would be that HVT infects melanocytes or their precursors, leading to their death and triggering an auto-immune response against melanocyte markers. In other genetic backgrounds, infection of these cells may have no impact on feather color and remain unnoticed. However, the ability of chicken melanocytes or their precursors to become infected by MDV has never been reported to date.

## 10. Cutaneous lesions after MDV infection

Macroscopic and microscopic lesions have been observed on the skin of infected chickens at the feather follicles or near them. Two types of lesions have been found: tumor-like and non tumor-like lesions. It is noteworthy that it was the tumor-like cutaneous lesions (often incorrectly called cutaneous leucosis) observed in the slaughterhouse that led to the initial suspicion that the skin was the main infected tissue in MD [73]. Birds presented hypertrophied feather follicles with compact lymphoid aggregates in the dermis associated with capillaries upon microscopic examination. The presence of MDV in tumor-like lesions was subsequently confirmed by isolation of the virus in culture [74]. However, in situ, these cells do not generally harbor viral antigens detectable by immunofluorescence [75], and therefore appear to be latently infected-tumor cells. Interestingly, cutaneous tumors with large accumulations of lymphoblasts expressing the viral oncoprotein Meq have been observed in the dermis of scaleless chickens, suggesting that the presence of feather follicles is not required for the development of skin tumors [70]. Among non tumor-like lesions are the nuclear inclusion bodies typically found during lytically herpesvirus infections. These nuclear inclusions are only found in the upper layers of the feather follicle epithelium, and never in the basal layer [42,45,75]. The lesions are associated with the presence of viral antigens. Analysis of the distribution of feather follicles positive for MDV antigens and lymphoid cell aggregates shows that these two features are associated [75], and suggests that lymphoid cells could be the source of feather follicle infection, although this has not been demonstrated. Macroscopic and microscopic lesions associated with the presence of MDV antigens have been described in cutaneous structures other than feather follicles, including the comb, barbs, and leg skin that harbors scales without feathers [76]. For more details on these skin lesions, we refer the reader to two reviews [20,77].

## 11. Atypical morphogenesis of MDV in the skin

All herpesvirus infectious particles have similar morphology, which consists of an icosaedric capsid containing the viral genome surrounded by the tegument and envelope. The particle, whose size differences depends mostly on the tegument’s thickness, is 200–250 nm in diameter for the type-species viruses, HHV-1 and PRV (pseudorabies virus) [78]. The particles are the result of a complex assembly, also termed viral morphogenesis, which follows three models. The most common model is that of envelopment-deenvelopment [79-83]. The assembly starts in the nucleus where the genome is incorporated in the capsids. These mature capsids, also called type C capsids, are then transported in the cytoplasm after budding at the inner nuclear membrane and fusion with the outer nuclear membrane. The envelopment-deenvelopment process creates a primary enveloped particle in the perinuclear space during the intermediate step. After reaching the cytoplasm, the capsids bind to tegument proteins and are reenveloped by budding into a membrane-bound organelle, probably the trans-Golgi network. Mature enveloped particles are then released in the extracellular medium by exocytosis. For the type-species alphaherperviruses HHV-1 or PRV, the number of mature viral particles in the cytoplasm and in the extracellular medium in various cell types is generally high. For MDV, mature viral particles are scarce in the cytoplasm (approximately 0.5% of total particles) [84] and have never been observed in the extracellular medium in cell culture. It is the same in the tissues of infected chickens, except skin (see review [85]). The skin has been shown to contain many MDV infectious particles in the epidermis of feather follicles in the transitional layer [42,86,87]. In these cells, particles are often within cytoplasmic inclusions constituted of electron-dense amorphous material and lacking visible peripheral lipid membranes. At higher magnification, enveloped particles located in these inclusions are 200–250 nm in diameter and do not seem to be surrounded by a second membrane [42], like predicted by the second envelopment process model. Because these two characteristics are atypical of alphaherpesviruses, they raise various hypotheses regarding the mechanism of final envelopment and excretion of the virus into the external medium. Are MDV virions excreted from the keratinocytes via active exocytosis or do they remain trapped in these cells until their final differentiation into corneocytes, and are they excreted passively in the environment by the physiological process of desquamation? These questions remain to be answered. To this date, there is no cell system that allows reproducing in culture the atypical viral morphogenesis observed in this tissue, which is a hindrance to its study as well as the study of other associated cellular determinants and processes.

As mentioned above, in 1970 the teams of Calnek and Nazerian were able to isolate viral particles from the skin of infected chickens and to observe them using TEM [41,42]. To this end, tissues were homogenized in water through freeze-thaw or by sonication. In these conditions, more than 50% of the viral particles observed were enveloped and had a diameter of 273–400 nm [41]. The size of these viral particles seems abnormally high compared to their estimated size in situ in the epidermis; therefore, this may be an artifact of the virus extraction method in hypotonic medium.

## 12. Viral molecules associated with MDV replication in feather follicles or with the infectiousness of particles excreted from the skin

Many studies have attempted to characterize the genes and/or viral proteins preferentially expressed in feather follicles in order to explain the high rate of morphogenesis observed in this tissue. A few viral proteins are expressed at a higher level in the feather follicle compared with that in other cell types in vivo or in culture. For instance, glycoprotein gD (encoded by the US6 gene) which is not usually expressed in chick embryo fibroblasts (CEF) in culture [88], is expressed in 30% to 50% of feather follicles positive for other viral antigens such as pp38 in experimentally infected chickens [61]. The role of gD expression in feather follicles is still unclear because the US6 gene is not required for MDV transmission between birds [89]. Tegument protein VP13/14 encoded by the UL47 gene is also strongly expressed in the epithelium of feather follicles of infected chickens, but is expressed weakly in the spleen and in CEF in culture [90]. However, the relationship between its high level of expression in the feather follicle and the high viral productivity in that tissue has not been investigated.

The major tegument VP22 protein encoded by UL49 also influences MDV horizontal dissemination. Indeed, fluorescent tagging of VP22 in C– or N-terminus abolished or diminished bird-to-bird transmission, respectively [14,90]. In the last case, the MDV genome copy number in feathers was reduced compared to the wild type [14].

To date, no cellular component has been found to be associated with the higher viral replication in feather follicles and specifically to its ability to produce a large quantity of infectious viral particles. The development of new molecular models of keratinocytes permissive to MDV infection in our laboratory may help solve this problem [91].

## 13. Excretion of vaccinating strains and pathogenic strains of MDV

The three currently available vaccines (HVT, GaHV-3 SB1, and GaHV-2 CV1988/Rispens) induce a non-sterilizing immune response which protects against tumor development. The Rispens strain is to date the best available vaccine against the most virulent strains of MDV. Because MDV is strictly associated with infected cells in culture, GaHV-2 vaccines are constituted of infected cells frozen in liquid nitrogen, a unique formulation for an antiviral vaccine. In poultry houses, vaccines are administered to 1-day-old chicks manually or in the embryo in ovo, 2–3 days before hatching using an automated injection system. All vaccinating strains replicate in feather follicles and their DNA is detectable in feather tips by qPCR [59,64]. The kinetics of detection of the genome of these strains is similar to that of pathogenic strains. For instance, the genome of the Rispens strains is detectable 4 to 7 days post-vaccination in the feather tips by qPCR [64,92], and the number of genome copies increases to reach 100-fold that measured in other tissues [64]. The number of copies of the Rispens genome at 21 days post-vaccination is highly variable between birds [93]. Whether quantitation of the genome of vaccinating strains in feathers by qPCR allows to evaluate the level of protection of the flock in poultry farms remains to be determined [93,94].

It is well established that vaccination does not block the infection of feather follicles by pathogenic strains and viral production, during experimental infection or in poultry farms [57,62,95]. In the past several years, qPCR methods were developed to discriminate between vaccinating strains and virulent strains. In particular, point mutation in the pp38 gene allowed to distinguish the attenuated Rispens strain from most pathogenic strains in the field [95]. The impact of vaccinating viruses on the replication of pathogenic viruses and vice versa is starting to be elucidated. Several studies have shown an increase in viral load for the HVT genome in feather after MDV infection, suggesting that infection by a virulent virus could increase the replication of the vaccinating virus [62,96]. This has not been observed with the Rispens homologous vaccinating strain [92]. The accumulation of the pathogenic strain RB-1B in feathers is reduced by approximately 10 times after vaccination with the Rispens strain, but its kinetics is not shorter (within the 21 days of the study) [92]. Nair hypothesized that vaccination allows pathogenic MDV strains to unobtrusively spread in poultry houses and could contribute to the evolution of viruses towards more virulent genotypes [17].

## 14. Immune response in the skin of MDV-infected chickens

Studies of the host immune response in feathers after infection with a highly virulent virus (like RB-1B) or by a vaccinating virus (like Rispens or HVT) show an increase in the expression of pro-inflammatory cytokine genes, particularly gamma-interferon, as well as an infiltration of CD4+ with or without CD8+ T lymphocytes [97,98]. The above mentioned results suggest that the immune response in feather follicles is relatively ineffective at blocking MDV replication in that tissue and at preventing its excretion in the environment. The cellular and molecular mechanisms that help protect against MDV replication and its excretion from the skin, are currently poorly characterized. Greater knowledge of these mechanisms would substantially help reduce the spreading of pathogenic strains in poultry houses.

## 15. Conclusions

In the past several years, the interactions between MDV and the skin have seen a renewed interest. Many studies have helped show that pathogenic viruses are excreted from feather follicles at high levels in the environment despite vaccination. The development of new techniques to measure the viral load from feather tips and dust has been essential to obtain these data. Blocking the excretion of pathogenic MDV is considered to date as a major goal to stop and prevent the evolution of MDV towards more pathogenic genotypes. Fundamentally however, many questions remain unanswered, particularly the molecular mechanisms and cellular components involved in the atypical morphogenesis of MDV in the epithelium of feather follicles leading to high production of infectious virions and environmental contamination.

## 16. Abbreviations

CEF, chicken embryonic fibroblasts; EGFP, Enhanced Green Fluorescent Protein; GaHV-3, Gallid herpesvirus 3; HHV-1, Human herpesvirus 1; HVT, Turkey herpesvirus (MeHV, Meleagrid herpesvirus); MD, Marek’s disease; MDV, Marek’s disease virus (GaHV-2, Gallid herpesvirus 2); mRFP, monomeric Red Fluorescent Protein; pi, post-infection; PRV, Pseudorabies virus (Suid herpesvirus 1); qPCR, quantitative PCR; TEM, transmission electron microscopy.

## 17. Lexicon

Barbs: Thick appendages located on both sides of the beak.

Bursa of Fabricius: Primary lymphoid organ specific of birds in which B lymphocytes are generated and selected. B lymphocytes exit from the bursa only at hatching. This organ, which is located on the dorsal side of the cloaca, regresses after 12 weeks of age and completely disappears.

Feather follicle: Region of the skin where a feather is formed and anchored (one follicle harbors one feather). The follicle ensures the renewal of the feather after a physiological or accidental loss.

Apteric skin: Area of the skin devoid of appendages (feathers, scales, etc.).

## 18. Competing interests

The authors declare that they have no competing interests.

## 19. Authors’ contributions

MC wrote the part related to chicken skin and feather follicles, prepared the figures and table. CD wrote the parts related to Marek’s disease virus. Both authors read and approved the manuscript.
